# The Roles of GRKs in Hemostasis and Thrombosis

**DOI:** 10.3390/ijms21155345

**Published:** 2020-07-28

**Authors:** Xi Chen, Xuefei Zhao, Matthew Cooper, Peisong Ma

**Affiliations:** 1 Cardeza Foundation for Hematologic Research, Department of Medicine, Sidney Kimmel Medical College, Thomas Jefferson University, Philadelphia, PA 19107, USA; Xi.Chen@jefferson.edu (X.C.); Xuefei.Zhao@jefferson.edu (X.Z.); Matthew.Cooper@jefferson.edu (M.C.); 2 Cyrus Tang Hematology Center, Soochow University, Suzhou 215123, China

**Keywords:** hemostasis, thrombosis, platelets, G protein coupled receptor (GPCR), GPCR kinases (GRKs)

## Abstract

Along with cancer, cardiovascular and cerebrovascular diseases remain by far the most common causes of death. Heart attacks and strokes are diseases in which platelets play a role, through activation on ruptured plaques and subsequent thrombus formation. Most platelet agonists activate platelets via G protein-coupled receptors (GPCRs), which make these receptors ideal targets for many antiplatelet drugs. However, little is known about the mechanisms that provide feedback regulation on GPCRs to limit platelet activation. Emerging evidence from our group and others strongly suggests that GPCR kinases (GRKs) are critical negative regulators during platelet activation and thrombus formation. In this review, we will summarize recent findings on the role of GRKs in platelet biology and how one specific GRK, GRK6, regulates the hemostatic response to vascular injury. Furthermore, we will discuss the potential role of GRKs in thrombotic disorders, such as thrombotic events in COVID-19 patients. Studies on the function of GRKs during platelet activation and thrombus formation have just recently begun, and a better understanding of the role of GRKs in hemostasis and thrombosis will provide a fruitful avenue for understanding the hemostatic response to injury. It may also lead to new therapeutic options for the treatment of thrombotic and cardiovascular disorders.

## 1. Introduction

Platelets are small, anucleate cells that circulate in the blood stream. They originate from megakaryocytes, which are produced from the myeloid progenitor lineage of hematopoietic stem cells. Megakaryocytes are polynuclear cells that reside in bone marrow cavities. Megakaryocytes in the vascular niche extend proplatelets and release them into vascular sinusoids. Once in the blood stream, these proplatelet structures branch and separate under flow stress, releasing proplatelet formations [1,2]. These proplatelets mature and decrease in size as they circulate, eventually becoming mature platelets. Platelets have a life span of 3–4 days in mice and 8–10 days in humans [3]. Under normal conditions, senescent platelets are cleared in the liver and spleen following platelet desialylation and phosphatidylserine exposure. Normal human platelet count is between 150,000 and 400,000 platelets per microliter of blood and 10^11^ platelets total are produced and destroyed per day in a healthy human [1,4].

**Figure 1:**
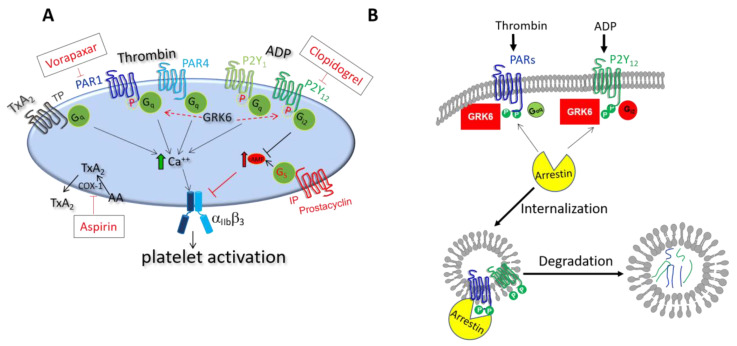
Feedback regulation of G protein coupled receptor (GPCR) signaling by GRK6 in platelets. (**A**) GPCR signaling in platelets. Many antiplatelet drugs target GPCRs signaling pathways, including PAR1 antagonist (vorapaxar), thromboxane A_2_ synthesis (aspirin), and ADP signaling (clopidogrel). *Left*: GPCR kinases (GRKs) are a critical negative regulator of GPCRs. (**B**) A model of GPCR desensitization and internalization in platelets. GRK6 regulates PARs and P2Y_12_-mediated signaling during platelet activation. In response to thrombin or ADP stimulation, GRK6 binds to PARs or P2Y_12_ in platelets and phosphorylates the serine residues of the receptor, leading to the desensitization of PARs or P2Y_12_. Arrestins are also involved in this process.

In circulation, platelets are vital for maintaining hemostasis. Upon detecting damage to the endothelial wall of blood vessels, platelets expose a variety of membrane receptors. Platelet surface receptors GPIb and GPVI bind to von Willebrand factor (vWF) and collagen, respectively, which are situated in a matrix behind the endothelium [5,6,7]. Additionally, thrombin released as a consequence of the coagulation cascade accumulates at the site of vascular injury and cleaves protease-activated receptors (PARs) on the platelet surface (Figure 1A). Initiation of these signaling events at the site of vascular injury leads to physical changes in the platelets. They undergo a change in shape, flattening and expanding their membrane surface and projecting pseudopodia. These signaling events also trigger integrin activation, which allows integrins on the platelet membrane surface to bind to fibrin/fibrinogen and causes platelets to link together [8]. Additionally, platelet activation leads to degranulation and the release of many signaling molecules stored in platelet α- and dense granules, including several growth factors, chemokines, vWF, ADP, ATP, calcium ions, and serotonin [9]. Furthermore, upon platelet activation, the platelet cyclo-oxygenase catalyzes the formation of thromboxane A2 from arachidonic acid [10]. Collectively, these signaling molecules form a rapid response mechanism that initiates platelet accumulation and thrombus growth at the site of injury. This thrombus “plugs” the hole in the vascular lumen, maintaining hemostasis. Using mouse models of hemostasis, platelet plug formation has been shown to be non-uniform [11,12]. The platelet plug “core” region, located near the site of injury, contains the most densely packed, fully activated platelets. Signaling in this region is dominated by thrombin (PAR receptor signaling). The platelet plug “shell” region contains relatively loosely packed and less activated platelets. Signaling here is dominated by thromboxane A2 (TP receptor signaling) and ADP (P2Y_1_ and P2Y_12_ receptor signaling).

## 2. GRKs in Platelets

Signaling that regulates platelet activation and accumulation largely occurs through G protein-coupled receptors (GPCRs) (Figure 1) [13]. Stimulation of G_q/12_-coupled receptors (PARs, TP, P2Y_1_), G_i_-coupled receptors (P2Y_12_), and G_z_-coupled receptors (adrenergic) generally lead to pro-activation signaling, while stimulation of G_s_-coupled receptors (IP) generally leads to inhibitory signaling. GPCRs activate G proteins by promoting the exchange of the GDP bound to the Gα subunit for GTP. Gα and Gβγ subunits then dissociate and stimulate their respective effectors (signaling “on”). There are several mechanisms that are in place to limit GPCR and G-protein dependent signaling in nucleated cells: (1) Receptors become desensitized to agonist stimulation upon phosphorylation by GPCR kinases (GRKs) and subsequent arrestin binding, which disrupts receptor-G protein signaling [14,15,16]; (2) Regulator of G protein signaling (RGS) proteins negatively regulate the α subunit of G proteins by increasing the rate of their intrinsic guanosine triphosphatase (GTPase) activity, thereby inactivating the Gα subunit and leading to the re-association of Gβγ (signaling “off”) [17,18], and (3) GRK2 binding to activated Gα_q_ (referred to as G_q_) inhibits further G_q_ signaling [19,20]. However, questions remain about the mechanisms by which GRKs provide negative feedback to activated GPCR in anucleate platelets. It is important to answer these as dysfunctional regulations of GPCRs can lead to pathological thrombus formation.

The canonical (GRK-mediated GPCR phosphorylation) and non-canonical (kinase-independent molecular interactions) function of GRKs in GPCRs regulation has been well-described and reported in many mammalian cell types. However, the presence and function of GRKs in platelets has been a more recent area of study. Messenger RNA and predicted protein copy number analysis in human and mouse platelets have identified the presence of GRK expression [21,22,23]. Further study using immunoblotting has confirmed the expression of specific GRKs (GRK2, GRK5, and GRK6) in platelets [24,25,26]. In human platelets, GRK2, GRK5, and GRK6 are all expressed in the range of 1000–2000 copies per platelet, with GRK6 having the highest expression level. Their expression in mouse platelets is similar, except for GRK5, which has a very low expression. GRKs have been extensively studied as potential targets in the development of novel therapeutic strategies in cardiac diseases. However, the function of GRKs during platelet activation and thrombus formation is still poorly understood. Given the critical role of platelets in hemostasis and cardiovascular diseases, there is a clear need to determine the functions of GRKs during platelet activation and thrombus formation.

## 3. The Role of GRKs and Arrestins during Platelet Activation

Platelet activation can be regulated at multiple places in its signaling network, including at the levels of receptor activation, intracellular Ca^2+^ elevation, RAP1 activation, and integrin outside-in signaling [27]. These different levels of regulatory events are essential to achieve optimal platelet signaling so that platelet activation is neither inadequate (allowing re-bleeding to occur) nor overly-exuberant (risking vascular occlusion). The first signaling node to control platelet activation after exposure to agonists is at the level of receptor stimulation. As mentioned above, one of the key regulators of GPCRs is the agonist-dependent phosphorylation by GRKs [28,29,30]. Phosphorylation of GPCRs leads to recruitment of arrestin, causing receptor desensitization and preventing further activation by de-coupling the G protein from the receptor. Phosphoproteome analysis shows that there is increased phosphorylation of Ser/Thr at the C-terminus of PAR-1 and PAR-4 upon platelet activation [31,32], which would promote recruitment of arrestin. Recently, there has been growing evidence of non-canonical activities for these kinases in nucleated cells, including kinase-independent molecular interactions and phosphorylation of non-receptor targets. These non-canonical activities have significant impact on cardiovascular function and disease progression [33,34]. The pivotal role of GRK2 and GRK5 in cardiac diseases is well documented. Cardiac GRK2 expression levels increase during hypertension, ischemia, as well as in early stages of maladaptive myocardial remodeling and heart failure [35]. On the other hand, the roles of GRKs and arrestins in platelets are just beginning to be uncovered.

### 3.1. Role of GRK6 during Platelet Activation

GRK6 was originally isolated in 1993 from the human heart cDNA library and identified as a new subtype of G protein-coupled receptor kinases [36]. GRK6 belongs to the GRK4/5/6 subfamily. It shares significant homology with GRK5 (70.1% amino acid similarity), β-adrenergic receptor kinase (GRK2) (37%), and rhodopsin kinase (GRK1) (47.1%) [36]. In both human and mouse platelets, GRK6 is the predominant form of GRKs. It impacts platelet activation through targeting of the PARs and P2Y_12_ receptors (Figure 1A) [25]. Human platelets express thrombin receptors, PAR1 and PAR4, but mouse platelets express a PAR3/PAR4 complex [37,38]. Kinetic studies in human platelets suggest that thrombin signals through PAR1 and subsequently through PAR4 [39,40]. Additionally, human PAR1 is cleaved and activated at both high and low concentrations of thrombin, while human PAR4 requires high concentrations [41]. Thrombin in mouse platelets is mediated by PAR3-faciliated cleavage of PAR4. PAR3 in mouse platelets acts as “cofactor” for PAR4-mediated thrombin signaling. Mouse PAR4 in complex with PAR3 leads to signaling at both high and low concentrations of thrombin, while stimulation of mouse PAR4 in the absence of PAR3 only produces platelet activation at high concentrations [42]. Thus, in the context of receptor signaling in response to thrombin stimulation, the function of mouse PAR4 is equivalent to that of human PAR1. That is, human PAR1 and the mouse PAR3-PAR4 complex display similar kinetic response upon agonist stimulation, while human PAR4 shows a delayed but sustained response. TxA_2_ activates platelets via TP receptor. In addition, human and mouse platelets express two distinct receptors for ADP, denoted P2Y_1_ and P2Y_12_.

#### 3.1.1. Regulation of Thrombin Receptor Signaling by GRK6

In response to thrombin stimulation, GRK6 binds to PAR1 in human platelets and phosphorylates the serine residues of the receptor, leading to the desensitization of PAR1. Using human megakaryoblastic cells (MEG-01), it has been shown that there is an increase in Ca^2+^ response to PAR1 agonist in GRK6^-/-^ cells, but no change in Ca^2+^ response to PAR4 agonist. This suggests that GRK6 regulates PAR1, but not PAR4, receptor-mediated signaling in human platelets [25]. Using CRISPR-Cas9 genome editing, we generated a GRK6 knockout mouse mutant line. We have shown that loss of GRK6 in mouse platelets increases both PAR4 and P2Y_12_-dependent signaling [25]. Notably, deletion of GRK6 has an effect on mouse PAR4-dependent signaling but not human PAR4-dependent signaling. Human PAR4 has been shown to internalize much less robustly than PAR1, likely because human PAR4 has fewer Ser/Thr phosphorylation sites than human PAR1 [39]. Furthermore, the C-terminus of human PAR4 has fewer Ser/Thr phosphorylation sites compared with mouse PAR4, and might not be targeted by GRK6. Taken together, GRK6 regulates PAR1-dependent human platelet activation and PAR4-mediated mouse platelet activation, respectively.

#### 3.1.2. Regulation of TxA_2_ Receptor Signaling by GRK6

TxA_2_ is generated from arachidonate in platelets by the aspirin-sensitive COX-1 pathway (Figure 1) [43]. TxA_2_ acts through TP receptors, which are encoded by a single gene. This gene can be alternatively spliced in the C-terminal tail, leading to two variants, TPα (343 residues) and TPβ (407 residues), which share the same first 328 amino acids. In nucleated cells, it has been shown that TPβ, but not TPα, undergoes agonist-induced internalization [44]. Notably, TxA_2_-stimulated effects in platelets are mediated predominantly through the α isoform [45]. We have shown that loss of GRK6 in mouse platelets does not affect TxA_2_-mediated signaling. This is because GRK6 mainly phosphorylates the C-terminus serine/threonine residues of GPCRs, such as TPβ, but cannot sufficiently phosphorylate the relatively shorter C-terminus of TPα. Therefore, GRK6 might not trigger TPα internalization and subsequent TPα-mediated signaling in platelets [25,26].

#### 3.1.3. Regulation of ADP Receptor Signaling by GRK6

Consistent with what we have documented, Hardy et al. also reported in astrocytoma cells that the desensitization of the P2Y_12_, but not P2Y_1_, is mediated by GRK2 and GRK6 [24], supporting the canonical function of GRK6 during P2Y_12_ receptor desensitization. Most recently, using GRK6 deficient mouse platelets, Chaudhary et al. reported that GRK6 is critical for regulating platelet activation through PAR4- and P2Y_12_-selective GPCR desensitization [26]. In contrast to the previous reports, they show that GRK6 might play a role in P2Y_1_-mediated signaling. Further studies are needed to validate the role of GRK6 in P2Y_1_-depedent signaling in platelets.

In addition, Chaudhary et al. demonstrated that GRK6 is not involved in the regulation of epinephrine α_2A_ adrenergic receptor- and serotonin 5HT_2A_-mediated platelet activation [26]. Our results suggest that GRK6 is not involved in the prostacyclin receptor (IP) signaling pathway [25]. Taken together, growing evidence from our studies and those from other groups reveal that (1) GRK6 plays a critical role during platelet activation and (2) GRK6 functions as a primary checkpoint to limit the intensity and duration of signaling for platelet activation via particular GPCRs.

### 3.2. Role of Arrestins during Platelet Activation

Termination of GPCR signaling requires not only the GRK-mediated phosphorylation of the receptors but also the function of arrestins, which are recruited and bound to activated GPCR. The activated GRCR/arrestin complex concentrates in punctate areas of the plasma membrane, where they co-localize with endocytic machinery like clathrin and AP2 [46,47]. In addition, arrestins can also function as an adaptor protein, which recruits various other proteins to promote arrestin-dependent signaling for different biological effects. It is reported that arrestins also act as scaffold proteins for mitogen-activated protein kinases (MAPKs) (including ERK1/2, p38, and c-JNKs), c-Src, PI3K, and Akt for various cellular functions such as cell cycle progression [48]. There are 4 different arrestins; arrestin-1 (visual arrestin), non-visual arrestin-2 (β-arrestin1), non-visual arrestin-3 (β-arrestin2), and cone photoreceptor specific arrestin-4 [49,50,51,52]. In platelets, arrestin-2 and arrestin-3 are the two major forms that are expressed. However, their functional contributions to platelet activation, hemostasis, and thrombosis have not been well studied.

A previous study by Schaff et al. using arrestin-2 and/or arrestin-3 knockout mice showed that platelet activation is not altered in the absence of arrestin-2 and arrestin-3 [53]. Furthermore, they conclude that arrestin-2 and arrestin-3 are not involved in P2Y_1_ and P2Y_12_ desensitization. Using both laser and ferric chloride injury models, Schaff et al. demonstrated that deletion of arrestin-2 in mice, but not arrestin-3, results in a decreased thrombus formation. They further revealed that arrestin-2 promotes thrombus formation through its participation in integrin α_IIb_β_3_ signaling, suggesting a direct signaling function of arrestin during platelet activation. It is still unclear whether arrestin-2 or arrestin-3 has functional redundancy in platelets for certain GPCRs, which contributes to the observed phenotype. Another study by Li et al. showed that the contribution of arrestins to PAR-mediated signaling has been limited to PAR4 [54]. PAR4-P2Y_12_ heterodimerization is involved in the recruitment of arrestin-2 to PAR4, where it has a positive signaling role. These two studies both indicate a direct signaling role for arrestin-2 instead of the expected desensitization of GPCR-dependent signaling in platelets. Whether the classical role of arrestin, the facilitation of desensitization of GPCR signaling observed in many cell types, exists in platelets remains elusive.

A recent study using arrestin-3 knockout mouse platelets has for the first time identified the negative regulatory function of arrestin-3 in platelets, which is demonstrated by increased platelet aggregation, secretion, integrin activation, and Ca^2+^ mobilization in response to some GPCR agonists in arrestin-3 knockout platelets, mainly downstream of PAR4- and P2Y_12_-mediated signaling pathways [55]. Overall, the gain of function phenotype in arrestin-3 deficient platelets indicates a negative regulatory role for arrestin-3 in limiting GPCR signal transduction.

Taken together, arrestin-3 regulates platelet activation via desensitizing PAR4 and P2Y_12_-mediated signaling (Figure 1B). Whether arrestin-2 plays a similar negative regulatory role in platelets still needs to be determined. In addition, identifying the scaffold function of arrestins in assembling signaling molecules/effectors during platelet activation would be an interesting avenue for future investigations.

### 3.3. Regulation of GRKs in Platelets

In cells other than platelets, GRKs are regulated from the moment they phosphorylate activated GPCRs [56]. Arrestin binding to phosphorylated GPCRs leads to clathrin-mediated endocytosis, which also internalizes the GRKs. Thus, GRKs in the internalized vesicles are prevented from further acting on other activated GPCRs. In addition to this regulatory mechanism, GRKs’ kinase activity can be modulated by multiple other mechanisms, such as interactions with calmodulin, caveolin, and actin, which fine-tune their kinase activities [57,58]. Among them, calmodulin is particularly of interest as it has been shown to regulate the function of several signaling molecules in platelets, such as PECAM-1, GPVI, and GP1b-IX-V [59,60,61]. A direct interaction between GRK5 and calmodulin has also been observed in other cell types. Both lobes of calmodulin bind with GRK5, which can inhibit its membrane association. The mechanism of GRK5 and GRK6 inhibition appears to be through the inhibition of GRK5 and GRK6 membrane association and subsequent receptor phosphorylation [62]. It has been shown that phosphorylation of receptors by GRK5 decreases, but GRK5 autophosphorylation increases, in a calmodulin-dependent manner [63]. Furthermore, this interaction leads to GRK5′s nuclear accumulation and potentiates NFAT and DNA binding, which enhances transcription of hypertrophic genes [64,65]. However, this mode of regulation via nuclear relocation is presumably not operative in anucleate platelets. How GRKs are regulated in platelets is an interesting area of study.

Achieving hemostasis following vascular injury while avoiding excessive platelet accumulation implies that GPCR signaling is closely regulated in both resting and activated platelets. It is reasonable to hypothesize that the function of GRKs is tightly regulated not only in activated platelets but also in resting platelets.

We and others have previously reported that the duration of G protein signaling in platelets is limited by RGS proteins and that removing the normal RGS-dependent limits on signaling produces a prothrombotic state [66,67,68,69,70]. The interaction between RGS proteins and G proteins in platelets is itself dynamically regulated by the presence of the scaffold protein spinophilin (SPL) [71]. Spinophilin was originally isolated as the binding partners of protein phosphatase 1 (PP1) and F-actin [72,73,74]. In resting platelets, spinophilin forms a novel tri-molecular complex in which spinophilin is bound to either RGS10 or RGS18, and the tyrosine phosphatase SHP-1. Platelet activation by thrombin or T_X_A_2_ activates SHP-1, leading to dissociation of the complex and release of RGS proteins to inhibit G protein-dependent signaling. Since GRKs contain a domain homologous to that of RGS, it is reasonable to propose that there is an interaction between GRK6 and spinophilin or calmodulin during platelet activation. It is possible that rather than competing for GRK protein binding, spinophilin and calmodulin could engage in a handoff during platelet activation that regulates the duration and magnitude of receptor signaling. Elucidating this mechanism of regulation in resting platelets and activated platelets would prove to be a promising avenue to investigate how GRKs can fine-tune the hemostatic response at site of vascular injury.

## 4. The Function of GRKs during the Hemostatic Plug Formation

### 4.1. Regulation of GPCR Signaling at Site of Vascular Injury

**Figure 2:**
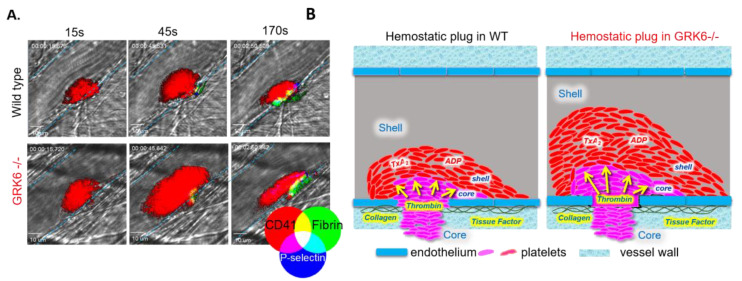
GRK6 regulates the hemostatic response to injury. (**A**) Increased platelet accumulation in GRK6-/- mice following laser injury in a cremaster muscle arteriole as described by Chen et al. [25] Platelets are labeled with fluorescently conjugated anti-CD41 (red), anti-Fibrin (green), and anti-P-selectin (blue). The blue lines mark the vessel walls. The pictures were taken 15, 45, or 170 s after injury. (**B**) The architecture of the hemostatic plug. Left: Hemostatic thrombi formed after penetrating injuries have a characteristic core/shell architecture in which the extent of platelet activation is determined by the distribution and concentration of agonists in the immediate environment of each platelet. The present studies and those discussed in the text show that the thrombus shell is dependent on ADP and TxA_2_, while the core requires thrombin. Right: Knocking out GRK6 causes an increase in PAR4- and P2Y_12_-mediated events, with a rapid increase of platelet accumulation during the early stage of thrombus formation.

Recently, a cremaster muscle arteriole injury model of the hemostatic response was established, in which the development of gradients of platelet soluble agonists present within the evolving platelet plug results in a gradient of platelet activation emanating from the injury site [11,69,75,76,77]. This injury model has one major advantage over other hemostasis/thrombosis models: the wealth of information obtained when coupled to intravital imaging provides a unique means to study the integration of molecular signaling pathways in vivo [78]. Briefly, in this laser-induced cremaster injury model, a nitrogen dye laser is used to produce a focal injury on the wall of 20–30 µm arterioles in the exteriorized cremaster muscle. Fluorescently tagged anti-CD41 F(ab)_2_ fragments, anti-P-selectin, and anti-fibrin antibodies are administered via a catheter in the jugular vein. Platelet accumulation and fibrin generation at the site of injury can then be detected in real time (Figure 2A).

Although initial adhesion of platelets to the vessel wall is driven by collagen, the subsequent recruitment of additional platelets into a growing thrombus requires mediators such as thrombin, thromboxane A_2_, and ADP, all of which act through GPCRs [13,79]. Studies in PAR4 knockout mice have demonstrated that thrombin signaling is critical for platelet accumulation in the cremaster laser injury model [80]. Recent studies also show that in the core region of the thrombus, thrombin mediates platelet activation with minimal requirement of ADP and TxA_2_. In contrast, ADP and TxA_2_ signaling are critical for outer shell region formation [11,77,78]. These observations have led us to propose that GPCR-dependent signaling may be tightly regulated in the growing platelet plug in order to achieve optimal response to injury.

### 4.2. GPCR Desensitization during Platelet Activation: An Old Question to Revisit

It has been long recognized that during platelet activation, GPCRs undergo desensitization following activation. For example, PAR1 undergoes rapid desensitization due to internalization, whereas activation-dependent internalization of PAR4 is much slower [39,81,82,83]. During platelet activation by thrombin or PAR1 receptor agonist peptide, two-thirds of PAR1 becomes internalized. However, approximately 40% of the cleaved PAR1 remain on the platelet surface [82]. In endothelial cells and fibroblasts, PARs, like other GPCRS, are internalized into endosomes. While other GPCRs, such as the β_2_-adrenergic receptor, are dephosphorylated and recycled to the membrane, PARs are targeted to lysosomes for destruction [84]. Subsequent to internalization and destruction of cleaved PARs, a new population of receptors is exposed on the cell membrane surface. Repopulation of the membrane surface with naïve receptors is independent of protein synthesis; the naïve receptors originate from an intracellular pool located near the membrane. In contrast to endothelial cells and fibroblasts, platelets lack the intracellular pool of thrombin receptors. This leaves platelets only able to respond to thrombin stimulation once, after which they are incorporated into the growing hemostatic plug. Clearly, there is a difference between anucleate platelets and nucleated cells, in the context of thrombin PAR1 receptor biology. Besides, platelets also become desensitized to activation upon continued exposure to ADP. It has long been shown that after being exposed to ADP, human platelets rapidly become unresponsive to a second stimulation with ADP [85]. Baurand et al. attempted to investigate the underlying mechanism of this unresponsiveness to ADP. They showed that P2Y_1_ and P2Y_12_ receptors are differentially regulated in this process. The P2Y_1_ receptor is rapidly desensitized and internalized, whereas the majority of P2Y_12_ receptor is still present on the plasma membrane and remains functional. They further proposed that even in platelets refractory to stimulation by ADP, the P2Y_12_ receptor is able to ensure platelet reactivity at the site of vascular injury [86]. In contrast to these findings, Hardy et al. showed that P2Y_12_ receptor undergoes desensitization in human platelets. Using a transfected cell-based assay, they further showed that GRK2 and GRK6 are involved in P2Y_12_ desensitization, while protein kinase C (PKC) regulates desensitization of P2Y_1_ receptor [24,87]. More studies will be needed to resolve these two contradictory results.

Interestingly, GPCR desensitization has generally been thought of as a late signaling event and functions as a “protective mechanism” in platelets. However, this statement has not been experimentally validated in vivo. In other cell types, GPCR desensitization can occur rapidly within seconds [88]. Collectively, further studies, such as using a systematic approach, are required to elucidate the consequence of GPCR desensitization in an evolving hemostatic plug.

### 4.3. The In Vivo Consequences of Deletion of GRK6 at Site of Vascular Injury

Using GRK6^-/-^ mice generated by CRISPR-Cas9, Chen et al. examined the consequences of GRK6 knockout on platelet activation and accumulation at the site of injury using the cremaster laser injury model. As discussed above, the thrombus architecture that forms consists of a core of tightly packed P-selectin positive platelets overlaid with a shell of loosely adherent P-selectin negative platelets. We found that platelets accumulate more rapidly at the site of injury in GRK6^-/-^ mice than in WT littermates (Figure 2A,B). Notably, Chen et al. demonstrated that the slope of platelet accumulation during the early stage of thrombus formation was increased in GRK6^-/-^ mice relative to WT controls [25]. This enhanced rate of platelet deposition resulted in increased total platelet accumulation in GRK6^-/-^ mice compared with WT mice. Thus, in contrast to the old paradigm of GPCR desensitization as a late signaling event, these recent results indicate that GRK6-mediated GPCR desensitization actually occurs as an early signaling event during platelet activation.

Using a ferric-chloride carotid artery injury model, Chaudhary et al. demonstrated that the time to occlusion of the carotid artery in GRK6 knockout mice is significantly shortened, suggesting enhanced thrombus formation in the absence of GRK6 in platelets [26]. Putting together these two injury models that rely on differently-sized arteries, we can see that deletion of GRK6 in platelets increases the magnitude and/or rate of platelet accumulation in the cremaster arterioles and increases the stability of occlusion in the carotid artery. This suggests that GRK6 plays a critical negative regulatory role during thrombus formation by limiting GPCR-dependent signaling.

## 5. GRKs in Human Pathology-Related Platelet Dysfunction

### 5.1. Antiplatelet Drugs Targeting at GPCRs and Their Regulators

Many antiplatelet drugs target GPCRs and their signaling pathways of platelet activation, including PAR1 antagonist (vorapaxar), thromboxane A_2_ synthesis (aspirin), and ADP P2Y_12_ signaling (clopidogrel, prasugrel, ticagrelor, and cangrelor). Besides the above reagents, a number of PAR1 and PAR4 antagonists are currently being evaluated in clinical trials [89]. In the past two decades, GRKs have been shown to play an important role in the heart by regulating GPCR signaling. Changes in GRK expression have been linked to many cardiovascular pathologies, including myocardial infarction, hypertension, and cardiac hypertrophy [90]. Therefore, GRKs have been extensively studied as therapeutic targets in cardiovascular disease. Although there is currently lacking pharmacologic studies on targeting GRKs in platelets, a better understanding of their role in hemostasis and thrombosis will be a key to the development of improved diagnostics and therapies for cardiovascular disease.

### 5.2. GRKs Polymorphisms and Their Role in Cardiovascular Disease

There is heritable interindividual variation in platelet reactivity that may be relevant to clinical events, such as myocardial infarction [91], and this heritability is higher in African American people than in Caucasian populations [92,93]. Recent studies have shown that SNPs in the human platelet PAR4 thrombin receptor contribute to a major fraction of the racial variance in PAR4-mediated platelet reactivity [93]. There is also sufficient evidence to suggest activated partial thromboplastin time (aPTT) is highly heritable [94]. aPTT measures the intrinsic and common coagulation pathway. A prolonged aPTT may be caused by congenital or acquired coagulation factor deficiencies. An abnormally reduced aPTT can indicate a hypercoaguable state in acute coronary syndrome. A study conducted from 2544 human subjects from the British Women’s Heart and Health Study shows that SNPs in GRK6 is associated with aPTT [95]. However, the impact of these SNPs on GRK6 expression and activity is unknown.

A non-synonymous SNP (rs17098707; T>A mutation) in GRK5 is associated with differential survival in African American heart failure patients [96]. In a study of 2673 acute coronary syndrome patients, improved outcomes were found in African American patients who had the GRK5 L41Q polymorphism. Another study on patients with coronary artery disease carrying L41Q suggested that the polymorphism was protective when they were treated for hypertension [97]. This polymorphism is also associated with left ventricular apical ballooning syndrome [98]. In addition to this L41Q polymorphism, two recent genome-wide association studies (GWAS) identified SNPs in GRK5 linked to platelet counts, mean platelet volume (MPV), and platelet volume distribution width (PDW) [93,99]. A recent genomic and transcriptomic association study showed that SNPs in GRK5 also associate with risk of venous thromboembolism (VTE) [100]. It would be interesting to examine whether these SNPs in GRKs mentioned above affect platelet activation and thrombus formation.

### 5.3. COVID-19 and Thrombotic Events: A Future Direction for Studying GRKs?

Currently, the novel coronavirus has affected many people worldwide and finding a cure is a top priority. Patients with COVID-19 have been found to face increased thrombotic risk [101,102,103]. Thrombotic events, in particular venous thromboembolism (VTE), have been shown to be present in some patients with COVID-19. VTE is a disease that includes pulmonary embolism (PE) and deep vein thrombosis (DVT), and platelets play a key role in initiating VTE [104]. Current data show that up to 15–39% of patients with COVID-19 infection who require mechanical ventilation have acute PE and DVT, and there is increased risk for VTE during this illness [105]. Furthermore, platelet activation in response to GPCR agonists is significantly increased in COVID-19 patients compared to healthy donors [106]. Several antithrombotic drugs have been proposed as potential therapies to prevent COVID-19 associated thrombosis, such as reagents targeting GPCRs, including a PAR1 antagonist (vorapaxar) [107,108,109]. However, the mechanism by which SARS-CoV-2 alters platelet activation/regulation to contribute to this prothrombotic state is not clear.

From a molecular point of view of the COVID-19 infection pathway, angiotensin-converting enzyme 2 (ACE2), to which the SARS-CoV-2 viral particles bind, is a critical component of the renin-angiotensin-system (RAS). ACE2 converts angiotensin II (Ang II) into Ang 1–7. These biological active fragments may subsequently activate the angiotensin II receptor type 2 (AT2). AT2 is a class of G-protein coupled receptors. Interestingly, the GRK-arrestin system negatively regulates AT2-mediated signaling [63,110]. Thus, it would be interesting to explore the molecular mechanism that could link altered GPCR regulation, platelet hyperreactivity, to thrombotic events in COVID-19 patients. Although these observations are still continuing to evolve, studies on the mechanisms by which GRKs contribute to thrombotic events will provide a valuable asset for patients care in the era of COVID-19.

## 6. Conclusions

In the past three decades, GRKs have been shown to play an important role in the heart by regulating GPCR signaling. Therefore, GRKs have been extensively studied as therapeutic targets in cardiovascular disease. However, there is a critical gap in knowledge between the unexplored function of GRKs in platelets and their well-studied role in cardiovascular health and diseases. The work published to date about GRK6 and arrestins in platelets may reveal only the “tip of the iceberg” of GRKs biology in hemostasis and thrombosis. Thus, ongoing studies may reveal meaningful information about 1) the role of GRKs in hemostasis and thrombosis and 2) the function of GRKs in platelets versus nucleated cell types. The insight gained from new approaches, such as genome-wide association studies (GWAS), expression quantitative trait loci (eQTL) analysis, CRISPR-Cas9 mediated genome-editing, and human iPSC cells will advance our understanding of 1) the role of GRKs in the cardiovascular system and 2) the effect of GRK genetic variants on platelet reactivity in general and in the context of racial differences. In summary, not only will these areas of investigation enable us to have a better understanding of GPCR regulation by GRKs, but they may also ultimately lead to new therapeutic options for the treatment of cardiovascular and thrombotic disorders.

## Abbreviations

GPCR	G protein-coupled receptor
RGS	regulator of G protein signaling
GRK	G protein-coupled receptor kinase
GTPase	intrinsic guanosine triphosphatase
PAR	protease-activated receptor
GP1b	glycoprotein 1b
GPVI	glycoprotein VI
VWF	von Willebrand factor
ADP	adenosine 5′-diphosphate
ATP	adenosine triphosphate
COX	cyclooxygenase
TXA2	thromboxane A2
IP	prostacyclin receptor
PECAM	platelet endothelial cell adhesion molecule
iPSC	induced pluripotent stem cell
MEG-01	megakaryoblastic cells
GWAS	genome-wide association studies
eQTL	expression quantitative trait loci
Ang II	angiotensin II
VTE	venous thromboembolism
ACE	angiotensin-converting enzyme
DVT	deep vein thrombosis
PE	pulmonary embolism
MPV	mean platelet volume
PDW	platelet volume distribution width
aPTT	activated partial thromboplastin time
PKC	protein kinase C
PP1	protein phosphatase-1
SPL	spinophilin
MAPK	mitogen-activated protein kinase
RAS	renin-angiotensin-system
WT	wild-type
